# Nucleation and Supercooling Mitigation in Fatty Alcohol Phase Change Material Emulsions for Heat Transport and Storage

**DOI:** 10.1021/acsomega.5c00041

**Published:** 2025-04-23

**Authors:** Moritz Kick, Sebastian Gamisch, Alexander Wittemann, Monika Le, Stefan Gschwander

**Affiliations:** † Fraunhofer Institute for Solar Energy Systems ISE, Heidenhofstr. 2, 79110 Freiburg, Germany; ‡ Colloid Chemistry, Department of Chemistry, University of Konstanz, 78457 Konstanz, Germany

## Abstract

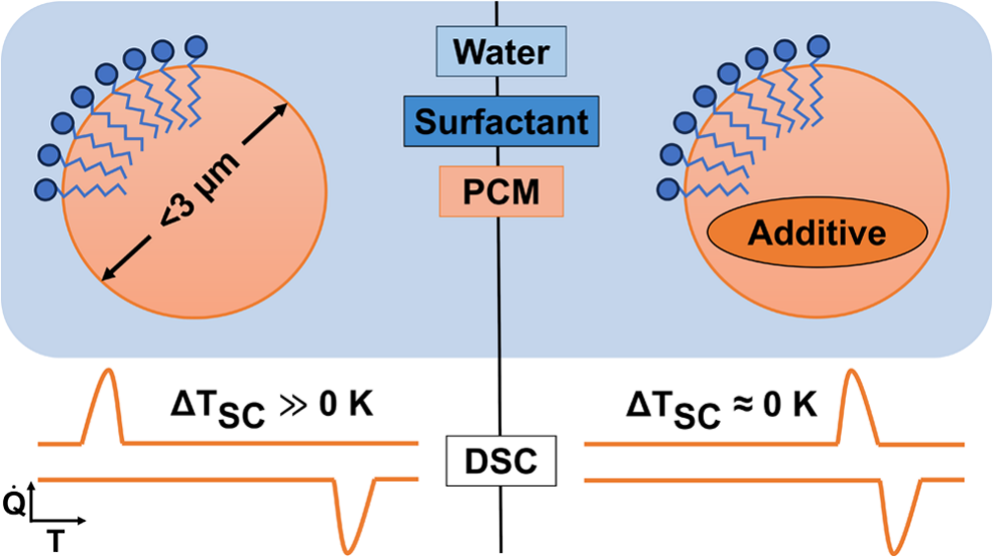

Organic phase change slurries (PCS) exhibit significant supercooling in small particles, which diminishes their advantages over sensible heat storage systems by reducing energy efficiency and reliability. While the mechanisms of supercooling in alkanes have been extensively studied, investigations of emulsions containing fatty alcohols are limited. This study examines the impact of nucleating agents on reducing supercooling in oil-in-water (O/W) emulsions of 1-docosanol, a fatty alcohol used as a phase change material (PCM). Emulsions with platykurtic particle size distributions were produced using rotor-stator emulsification. Various material combinations were explored to identify nucleation promoters compatible with 1-docosanol. Thermal analysis revealed that long-chain polymers effectively mitigate supercooling, particularly during rotator phase transitions, as confirmed by crystal structure analysis. On average, supercooling was reduced by 9 K; however, seed deactivation observed over multiple thermal cycles led to a gradual return of supercooling. Similar to alkanes, particle size influences the nucleation rate, surfactants affect the availability of heterogeneous nucleation sites, and nucleating agents can decrease the nucleation barrier. The findings indicate that high structural similarity between emulsion components is beneficial for minimizing supercooling in PCS, enhancing their potential for thermal energy storage applications.

## 1 Introduction

Phase change materials (PCMs) have attracted considerable attention as promising alternatives to conventional sensible thermal energy storage systems due to their high latent heat storage capacity.^1−4^ Supercooling occurs when a material is cooled below its melting point without undergoing solidification, thereby extending the PCMs operating temperature range.^5,6^ This can negatively impact the efficiency and reliability of latent heat storage applications across various sectors, including transportation, food processing, medical sciences, and air conditioning.^7−21^ Consequently, mitigating supercooling is a crucial research area for PCMs such as organic waxes.^22^ Organic phase change material emulsions (“PCME”; or slurries, “PCS”; sometimes dispersions, “PCMD”) are colloidal systems that allow for efficient heat transfer and transport, but are often limited by the presence of supercooling, which diminishes these advantages.^23−26^ Reducing supercooling in organic PCS enhances their thermal properties and reduces the energy consumption of these systems.^27−33^ Supercooling arises from the absence of nucleation and becomes more pronounced as the particle size of the PCS decreases. Nonetheless, small particles are required to increase emulsion stability against creaming and maximize surface area for rapid heat exchange. Finite-size effects have been observed in particles smaller than approximately 3 μm, and supercooling depends on several factors besides the PCM itself.^34−37^ Significant preliminary work on supercooling in *n*-alkane PCS has been conducted by various research groups, who have also evaluated different nucleation mechanisms.^31,34,38−46^ A common finding is that homogeneous nucleation in isolated small particles definitively causes supercooling; however, calculated nucleation rates suggest a mechanism involving slow heterogeneous nucleation instead.^34,47,48^ Surfactants have been reported to influence these nucleation rates,^49,50^ with structural similarities between the PCM and the lipophilic part of the surfactant reducing supercooling.^28,32,51−53^ Additionally, numerous authors propose the intentional use of specific additives to promote heterogeneous nucleation.^33,42−44,54^

Nucleating agents have been identified as a promising solution for reducing supercooling in oil-in-water (O/W) PCS by providing heterogeneous nucleation sites for crystal growth. The effectiveness of these agents, or seeds, depends on their ability to promote nucleation, which is influenced by their size, shape, and chemical composition. Commonly employed seeds include metal particles, inorganic salts, or other PCMs from the same material class with higher melting points than the target PCM.^55−59^ In paraffins, metastable rotator phases occur between the liquid state and the thermodynamically stable crystalline phase, leading to additional solid–solid transitions at lower temperatures, each with its own degree of supercooling.^60−69^ This phenomenon partially shifts the available phase change enthalpy away from the temperature range relevant for temperature sensitive applications like food preservation. These transient rotator phases are analogous to liquid crystals, allowing rotation around the principal axis of molecules that are otherwise ordered.^70,71^ To date, studies on the influence of nucleation additives have focused exclusively on the liquid to high-temperature rotator phase (RII) transition, as this transition is primarily affected by supercooling and releases the majority of the stored heat.^29,32,72^ The lower temperature transitions are typically neglected, even though they contribute to the latent heat storage potential. Therefore, the aim of this investigation is to shift all phase transitions during cooling to the bulk melting temperature. Since melting temperatures represent the thermodynamic stability limits of a phase, the key question is whether and how all transitions can be shifted back to the bulk melting temperature.

The presented approach for nucleation support with additives focuses on structural similarities between the PCM and the seed. The seeds can be either soluble or insoluble. Soluble seeds separate during cooling by spinodal decomposition from a mixture of similar materials while insoluble seeds exist permanently as a solid particle. The structural similarity is supposed to increase compatibility of the seed surface with the PCM molecules, so that the energy barrier for crystal growth is as small as possible. There are a few publications using the same approach of higher homologs in other materials^73,74^ including our preliminary work on 1-docosanol nucleation.^75^ Graswinckel et al. showed epitaxial growth of *n*-alkane crystals on graphite^76^ giving proof for the feasibility of such an approach. Reliable observation of supercooling in organic phase change dispersions is essential for exploring the fundamental principles of this phenomenon and for developing effective mitigation strategies.^72,77^ To achieve supercooling in particles, dispersions of the fatty alcohol PCM 1-docosanol (C22-OH), water and surfactants are used. Surfactants Laureth-2, Laureth-30, Ceteth-80, Poloxamer 407 and Inulin Lauryl Ester (Table S1) and seed materials C50-OH, C70, ZnO, Carbon black and CuO (Table S2) are investigated based on their structure and properties to identify their potential for supercooling mitigation. The surfactants are selected according to the Hydrophilic–Lipophilic Balance (HLB) concept.^78^ The HLB describes the surfactant (and their combinations) in terms of hydro- or lipophilicity serving as a key indicator of material compatibility. For O/W dispersions, a surfactant mixture with an HLB value between 8 and 18 is suggested.^79−81^ Since the HLB is not defined for polymer surfactants, they are selected based on literature recommendation.^82^ Laureth-2 is used in combination with Laureth-30 to adjust the mixture HLB, while the other surfactants are used on their own in different concentrations between 2 and 6 wt %.

## 2 Results and Discussion

**Figure 1:**
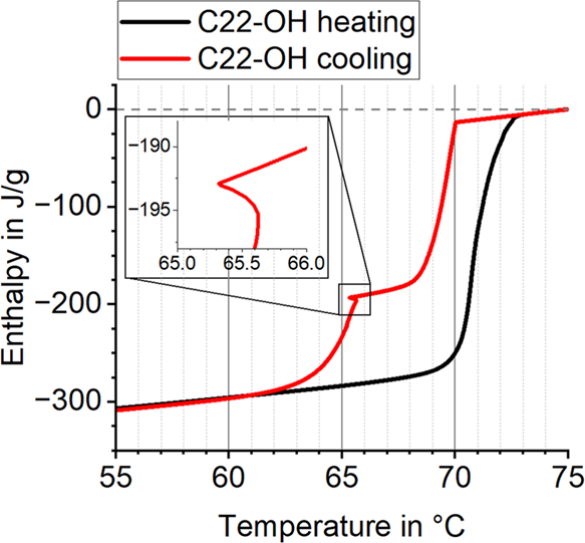
Melting and crystallization enthalpy curves of 1-docosanol, illustrating differences due to a combination of supercooling and hysteresis. Supercooling is identified by temperature rises during crystallization.

Emulsion production involved independently preparing the oil and water phases by dissolving their respective components under continuous stirring. These phases were then combined to form a preliminary emulsion, which was subsequently dispersed and homogenized to yield the final emulsion. Thermal characterization and stability assessment was performed using thermal cycling analysis between 20 and 80 °C with differential scanning calorimetry (DSC). A heating/cooling rate of 1 K/min was selected because it balances providing the sample sufficient time to achieve thermal equilibrium with completing the experiment in a reasonable time. At this rate, thermal gradients within the sample are minimized, thereby reducing kinetic artifacts such as thermal lag and nonequilibrium phase transitions. The enthalpy curve of pure 1-docosanol (Figure 1) exhibits linear regions at the beginning and end, representing sensible heat, while the steep intermediate sections correspond to latent heat storage and release. The total melting enthalpy including both latent and sensible heat of C22-OH is 284.7 ± 5.7 J/g. The difference between melting and crystallization curve results from a combination of supercooling and hysteresis, which are difficult to separate quantitatively. Supercooling can be identified by the small temperature rise from sudden heat of solidification release at around 65 °C (red curve). This temperature increase is frequently observed in PCM crystallization processes due to physical device limitations that cannot dissipate the high latent heat released. It can also be reproduced with different heating rates because the latent heat release is not influenced by the measurement settings. Ultimately, this leads to an ambiguous measurement curve with temperature increase. From a physical perspective, a higher heat flow would be expected instead. Furthermore, this does not imply the absence of supercooling in other regions. Instead, it may be obscured by hysteresis and physical limitations such as limited heat flow.^83^

**Figure 2:**
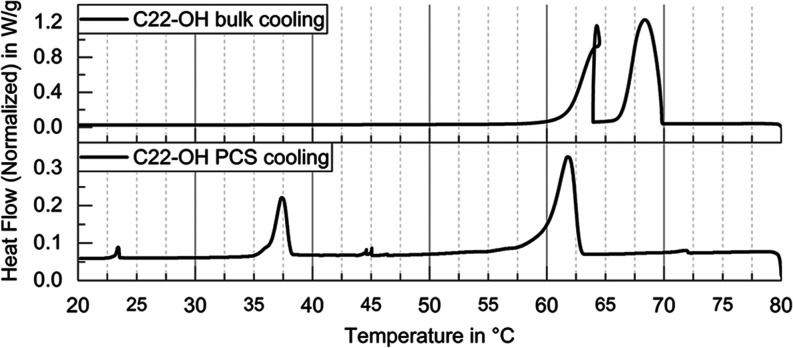
DSC cooling heat flow diagrams of bulk 1-Docosanol (top) and the PCS (bottom) with Laureth-2 and Laureth-30 (exo up). Sudden enthalpy release from the supercooled rotator phase in bulk leads to a slight temperature increase (loop).

**Figure 3:**
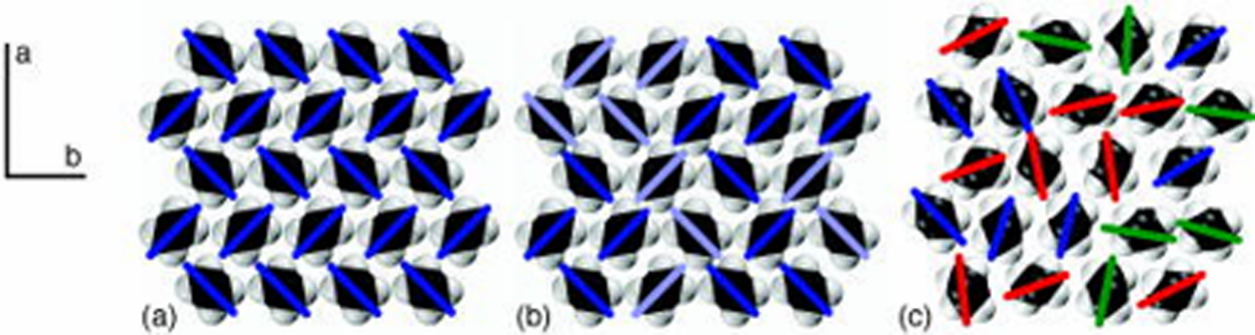
“Cross sections of three different *n*-alkane solid phases as viewed down the molecular c axis with the a and b axes as shown. Colored lines show the orientation of the molecules (the average projection of the C–C bonds onto the a-b plane). (a) shows an orthorhombic herringbone crystal. (b) shows an orthorhombic RI phase. The light lines show molecules that are turned by 90° from (a). (c) shows the hexagonal RII phase. Molecules remain in layers but are oriented in various directions” with varying degrees of rotational freedom observed in 1-docosanol. Reprinted with permission from Wentzel et al.^85^

Dispersions containing 20 wt % C22-OH, surfactant and water were used throughout this study. To optimize the dispersions, various surfactants and their combinations were screened as previously described. Because effective stabilization minimizes surface energy and leads to smaller droplets, the d90 value of the particle size distribution was used for evaluation, measured by static light scattering with Mie theory. The dispersions explored in this study with small particle sizes exhibited a supercooling increase similar to that reported previously.^40^ A quasi-monomodal particle size distribution below 1 μm (d90 of 0.39, standard deviation of 0.12 μm, kurtosis of −0.23; Figure S1) and consistent supercooling behavior in the DSC analysis was achieved using a PCS formulated with 20 wt % fatty alcohol C22-OH, a total of 6.0 wt % Laureth-2 and Laureth-30 mixture with HLB 16.6, and 74.0 wt % deionized water. This formulation produced the highest degree of supercooling among the examined emulsion compositions (Table S3) and therefore was used for further investigations into supercooling reduction with nucleating agents. Figure 2 presents a comparison of the exothermic DSC heat flows during crystallization for bulk C22-OH and the stable C22-OH dispersion. The bulk material exhibits two distinct transitions with onset temperatures at 69.8 and 63.9 °C, corresponding to the rotator phase transitions described by Sirota et al. and Wentzel et al. (Figure 3). The reported bulk values^82^ match the here presented measurements within variance: the liquid to rotator phase transition releases 163.9 ± 3.5 J/g of latent heat and the rotator phase to solid transition releases 82.6 ± 1.7 J/g of latent heat equivalent to a 2:1 ratio.

**Figure 4:**
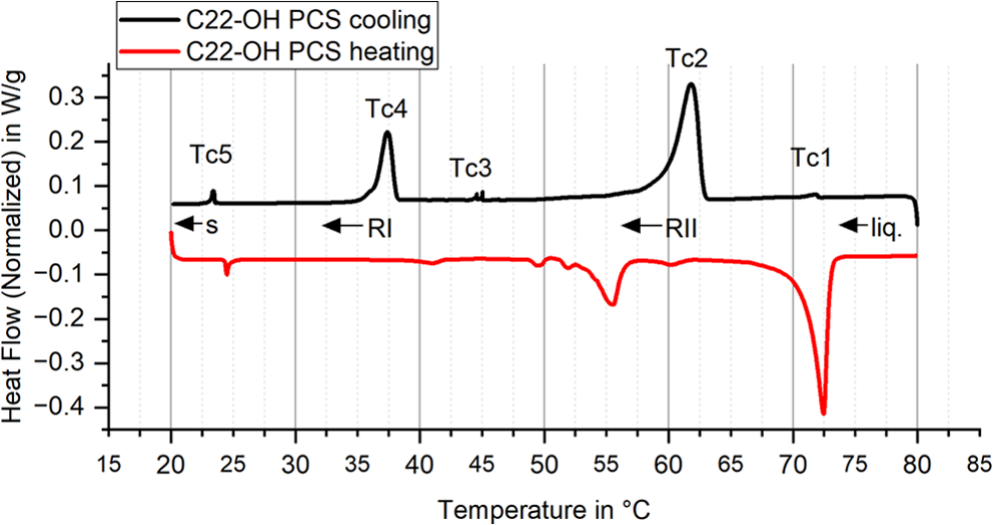
DSC heat flow diagram of the PCS without seed, including the cooling and heating curve (exo up). The peaks and expected phases during cooling are labeled according to the simulations of Wentzel et al.

The bulk material does not show an additional low temperature transition to the metastable rotator phase 1 (RI) in contrast to the expectations from Sirota’s reports.^82^ This RI phase typically appears only in dispersions, along with a general decrease in transition temperatures (Figure 2, bottom). For the bulk material, this suggests that several transitions overlap and cannot be distinguished. Figure 4 shows the melting and crystallization heat flow curves for the dispersions, where Tc*n* denotes the temperature of transition *n* during cooling. Both the described rotator phases RI (low temperature, Tc4) and RII (high temperature, Tc2) are presumably attributed to the respective transitions. The DSC also reveals additional small peaks, one at 72.1 °C (Tc1) close to the bulk melting temperature that indicates PCS crystallization at higher temperatures is possible, and one at 45.1 °C (Tc3) that apparently belongs to one of the rotator phases. The lowest temperature transition at 23.6 °C is likely the formation of the stable crystal phase s (Tc5) which is significantly shifted.

To maximize the available enthalpy at the bulk melting temperature, nucleation seeds are used. The enthalpy ratio ER of a dispersion is used as evaluation criteria for the effectiveness of the seed in mitigating supercooling. The ER is calculated by the fraction of total phase change enthalpy *H* released at the (bulk) melting temperature (Tc1) with eq 1 and emphasizes the significance of the initial transition, although the other transition enthalpies are calculated accordingly. In this case, the emulsion exhibits significant supercooling, resulting in an ER of 1.1%.
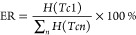
1

The significant peaks on the heating curve match those on the cooling curve in terms of enthalpy but differ in temperature. According to the enthalpies, Tc2 corresponds to the melting peak at 71.1 °C (close to Tc1). Tc4 and Tc3 transition together at 53.5 °C (including the small peaks before at 41 and 49 °C) and Tc5 transitions at 24.1 °C. This indicates a relationship between the transitions and allows for the calculation of relative supercooling values as the difference between the onset temperature of the peaks on the cooling curve and onset of the next higher transitions on the heating curve. Absolute supercooling is calculated with respect to the corresponding melting onset of Tc1. These values allow for a detailed discussion of seed impact later. Negative values may occur due to the method of calculating supercooling Δ*T* as the difference in onset temperatures of melting and crystallization, shown in eq 2.
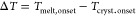
2The liquid-to-RII transition at 62.9 °C on the cooling curve is supercooled by 8.2 K compared to the melting onset of 71.1 °C and contains 72.1% (40.3 J/g) of the latent enthalpy. The RII to RI freezing onset of 38.2 °C has a much lower temperature as compared to the next melting onset at 53.5 °C (omitting the small peak before due to negligible enthalpy), resulting in a relative supercooling of 15.3 K and an absolute supercooling of 32.9 K. It contains 23.3% (13.0 J/g) of the latent enthalpy. Compared to the bulk material (compare Figure 2) this is not matching the previous 2:1 ratio of the phases but rather a 3:1 ratio. The RI to crystal (s) transition at 23.4 °C is very close to its melting counterpart, with 0.9 K relative supercooling. Still, it is 47.7 K below the melting onset temperature for Tc1 and 1.5% (0.8 J/g) of the latent enthalpy. The average results over three experiments are summarized in Table 1. The discrepancy in the phase ratio exceeds the calculated error margins. This indicates that the change in phase ratio is a real effect potentially attributable to the influence of emulsification on the crystallization behavior. Additionally, the total latent heat of the emulsion sums up to approximately 23% of the bulk latent enthalpy, where only 20% are expected. This is explained by water evaporation during manufacturing, whereas C22-OH does not evaporate.

**Table 1:** Onset Temperatures of the PCS Phase Transitions without Seed during the First Cooling Ramp with Latent Heat and Their Relative and Absolute Supercooling Values (Average over Three Experiments with Standard Deviation)

transition	onset temperature in °C	latent enthalpy in % of total	relative supercooling in K	absolute supercooling in K
Tc1	72.0 ± 0.7	1.1 ± 2.7 (ER)	–1.0 ± 0.2	–1.0 ± 0.2
Tc2	63.1 ± 0.3	74.8 ± 4.2	7.9 ± 0.5	7.9 ± 0.5
Tc3	45.1 ± 0.5	0.2 ± 2.5	11.3 ± 2.9	25.9 ± 0.2
Tc4	38.3 ± 0.3	23.7 ± 4.4	18.1 ± 3.1	32.7 ± 0.4
Tc5	23.6 ± 0.2	1.2 ± 2.1	0.9 ± 0.1	47.4 ± 0.2

### 2.1 Paraffin Seed Impact

**Figure 5:**
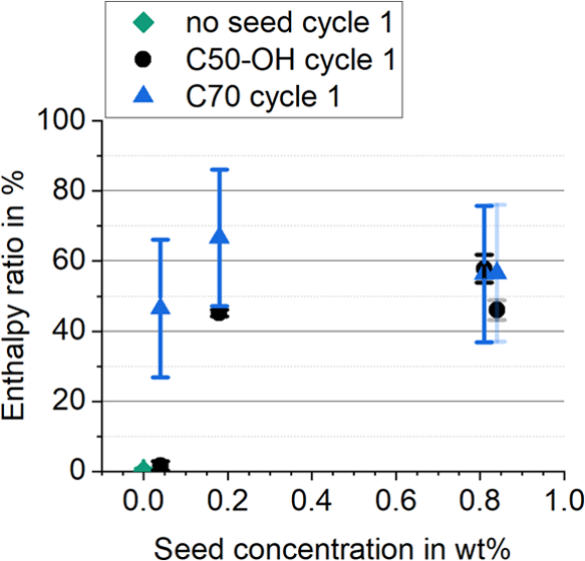
Enthalpy ratios of PCS with different concentrations of C50-OH and C70 in the first thermal cycle. Error bars show the standard deviations over multiple samples (bold) or approximated values from averages of all samples if only one measurement at the respective seed concentration is available (light).

A perfect seed is supposed to shift all enthalpy from transitions (Tc*n*, *n* > 1) at lower temperatures to the highest (bulk) transition (Tc1) temperature, thereby increasing the ER. The PCM C22-OH has an orthorhombic crystal structure,^83^ prompting tests with seed materials of similar structure. Experimentally, the seed amounts were adjusted so that their separation temperature exceeded the PCM melting temperature, while remaining below the manufacturing temperature to ensure proper mixing. Adding the orthorhombic seed C50-OH to the dispersion at different concentrations increases the ER, starting from 0.18 wt % (Figure 5). C70 exhibits a strong impact even at 0.04 wt % surpassing C50-OH at 0.18 wt %, but otherwise behaves similarly, with an ER between 40 and 60%. The stronger effect of C70 at low concentrations is likely caused by the lower miscibility with the PCM, leading to earlier seed formation by spinodal decomposition as compared to C50-OH. At higher concentrations, the ER does not significantly increase beyond the effect at 0.18 wt % in thermal cycle one. This shows that nucleation can be triggered with minimal additive. A statistical distribution throughout PCS droplets can be assumed.

The standard deviations in Figure 5 are calculated across different experiments (bold error bars) or approximated (light error bars) when only one respective sample was available. The approximation is based on the average standard deviation for a PCS, assuming codependent enthalpy shifts of similar magnitude between transitions. The standard deviations of different C70 samples with the same seed suggest a significant impact of random nucleation events, indicating that variations in C70 concentrations may be due to chance. In contrast, C50-OH results are more consistent across multiple experiments, likely due to its high structural compatibility with the PCM. However, the ER unexpectedly decreases at the highest reported concentration. The particle sizes (d90) of all PCS considered are within a 0.42 ± 0.16 μm range, excluding a strong impact of particle size alone. This observation may be attributed to the manufacturing process not being accounted for in the thermal analysis.

**Figure 6:**
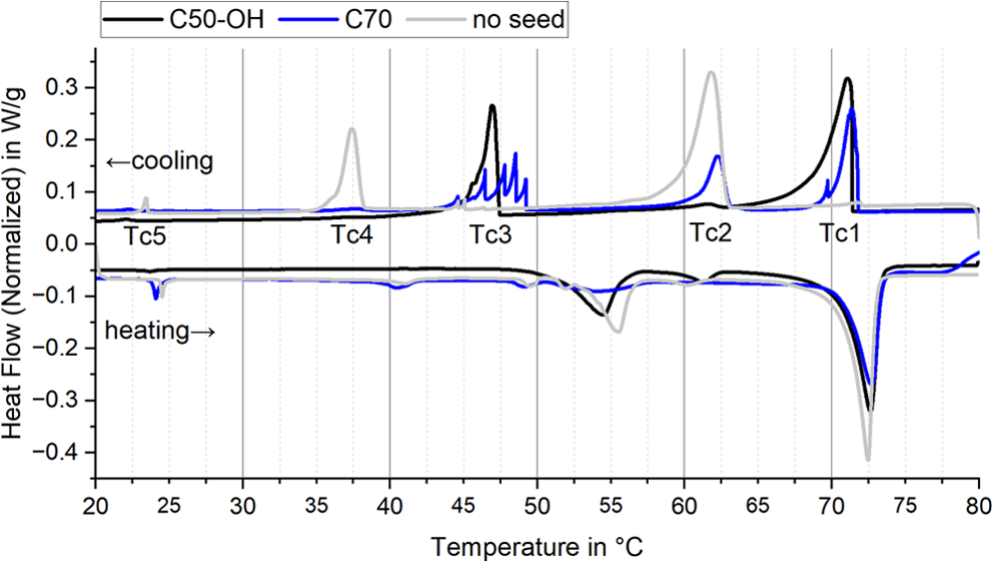
DSC heat flow diagrams of dispersions with 0.8% seed in thermal cycle one. C50-OH shifts the crystallization peaks almost completely (black), C70 partly shifts them and shows irregular peak patterns related to different particle sizes (blue). The melting transitions are very similar, even with the reference dispersion without seed (gray).

Figure 6 compares the heat flow curves for melting and crystallization of PCS with 0.81 wt % seed C50-OH or C70 to a PCS without seed. With C50-OH, the liquid to RII onset (Tc1) shifts from its supercooled position at 62.9 °C to its bulk temperature of 71.7 °C increasing the ER from 1.1% to 58.8%. A small fraction of the supercooled peak remains, likely due to the absence of C50-OH inside the smallest droplets. Statistically, each droplet contains approximately 10^17^ seed molecules, but fluctuations may lead to undercutting of a seed formation threshold in the smallest droplets. The RII to RI transition (Tc4) shifts completely from 38.2 to 47.4 °C (Tc3) with a remaining relative supercooling of 3.9 K and absolute supercooling of 23.3 K. Both transitions overlap with the small peaks (Tc3) present in the original dispersion without seed (Figure 6, gray curve). The RI to solid transition (Tc5) is barely affected, with a supercooling decrease from 1.1 to 0.6 K.

This behavior is reproducible (Table 2), although the enthalpies for Tc2 and Tc4 occasionally shift between the corresponding enthalpies for Tc1 and Tc3, respectively. Consequently, C50-OH effectively supports phase transitions, nearly eliminating supercooling in the liquid to RII transition (Tc2), resulting in an ER of 58.0 ± 4.7%, and reducing it by approximately 13 K in the RII to RI transition (Tc4), resulting in 20.0 ± 19.0% latent enthalpy. The melting peaks observed in DSC (Figure 6) are minimally affected by the seeds, suggesting that they occur at a thermodynamically stable temperature and, as a result, lower temperature crystallization events must be supercooled. The endothermal transitions at 71 and 24 °C remain unchanged, while the transition at 53 °C is broadened and shifted. Each of the melting peaks corresponds to a crystal phase the PCM undergoes. The small peaks around 40 °C, 50 °C (no seed/C70) and 60 °C (C50-OH) are unexpected but can be attributed to the Gibbs–Thomson effect in small particles.

**Table 2:** Onset Temperatures of the PCS Phase Transitions with Seed C50-OH during the First Cooling Ramp with Latent Heat and Their Relative and Absolute Supercooling Values (Average over Three Experiments with Standard Deviation)

transition	onset temperature in °C	latent enthalpy in % of total	relative supercooling in K	absolute supercooling in K
Tc1	71.0 ± 0.9	58.0 ± 4.7 (ER)	–0.2 ± 1.2	–0.2 ± 1.2
Tc2	63.1 ± 1.4	15.5 ± 6.1	7.8 ± 1.8	7.8 ± 1.8
Tc3	50.5 ± 4.2	20.0 ± 19.0	2.4 ± 7.6	20.4 ± 4.5
Tc4	37.6 ± 0.5	4.0 ± 9.0	15.3 ± 3.9	33.3 ± 0.8
Tc5	22.8 ± 0.6	2.5 ± 5.9	0.7 ± 1.0	48.1 ± 1.0

Using C50-OH, the crystallization peaks are shifted to higher temperatures, but only the RII phase returns to its original phase transition temperature. Since the respective phase transition during heating usually reflects the thermodynamically highest stable temperature of a phase,^87,88^ it is questionable whether the other peaks can be shifted to the bulk temperature in microcompartments at all, or if they reach their maximum at the corresponding melting temperature.

Changing the seed to C70 yields similar temperature results (Table 3, Figure 6 blue curve), but the enthalpy distribution differs. The enthalpy shift to Tc1 is slightly weaker than for C50-OH with 18.9 ± 12.6% remaining at the supercooled Tc2 on average. In contrast, the standard deviation between related peaks Tc1 and Tc2 is much greater than for C50-OH, likely due to more frequent enthalpy shifts. This could result from C70 being less compatible with 1-docosanol making nucleation less reliable and more dependent on the statistical nature of nucleation itself. Tc3 and Tc4 show similar behavior, while the irregular multiple-peak shape of Tc3 is a typical sign of independently crystallizing dispersion droplets.^51,84,85^

**Table 3:** Onset Temperatures of the PCS Phase Transitions with Seed C70 during the First Cooling Ramp with Latent Heat and Their Relative and Absolute Supercooling Values (Average over Three Experiments with Standard Deviation)

transition	onset temperature in °C	latent enthalpy in % of total	relative supercooling in K	absolute supercooling in K
Tc1	70.9 ± 0.9	55.8 ± 21.4 (ER)	0.6 ± 1.2	0.6 ± 1.2
Tc2	62.2 ± 0.8	18.9 ± 12.6	9.2 ± 1.1	9.2 ± 1.1
Tc3	48.0 ± 1.1	9.5 ± 18.5	5.7 ± 4.9	23.5 ± 1.4
Tc4	37.6 ± 1.1	11.1 ± 12.2	16.1 ± 4.9	33.9 ± 1.4
Tc5	23.2 ± 0.7	1.2 ± 3.1	0.8 ± 1.2	48.3 ± 1.0

Using either seed material leads to a high standard deviation for the peaks Tc3 and Tc4, which sometimes show only one transition, though the reason is unclear. Tc3 and Tc4 are solid rotator phase transitions that do not need nucleation in the traditional sense, yet the transition is shifted by the seed material. This could depend on the seed crystallite size, where a small seed suffices for the liquid-to-solid transition, but a larger crystallite is necessary for the seed to change rotator phases. Large seeds without intercalated PCM molecules can then offer a surface for rotator phase orientation and lower the switching barrier. Since the results of other transitions and PCS deviate much less and repeated measurements yield similar results, a systematic measurement error is unlikely. A limitation could be slight variations in manufacturing and sample waiting times before measurement.

### 2.2 Nanoparticle Compatibility

Nanoparticle seed compatibility is based on the similarity of crystal structures between the PCM and the additive. Therefore, it is crucial to compare the geometric parameters (Table S2 lists space group, lattice parameters *a, b, c* and angles α, β, γ). These nanoparticles must be smaller than the dispersion droplets to trigger the crystallization of the PCM they are enclosed in. 1-docosanol has the Space Group A 2/a (Nr. 15) and lattice parameters *a* = 9.00 Å, *b* = 4.98 Å, *c* = 118.58 Å, α = γ = 90 ° and β = 122.51 °.^86,87^ Comparing the *b*-parameter to the nanoparticles, it is close to the CuO *c*-parameter of 5.13 Å and its *a*-parameter of 4.69 Å. These, along with the *c*-parameter of ZnO with 5.21 Å, are less than 7% off the 1-docosanol *b*-parameter. Smaller values require fewer crystal defects during growth and should be more effective in nucleation. The *c*-parameter of 1-docosanol was interpreted differently as the distance between two carbon atoms pointing in the same direction (1,3-distance due to tetrahedral geometry) and was calculated to be 2.51 Å. The lattice mismatch parameter *f* is calculated by eq 3.
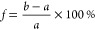
3Calculating each of the possible combinations (Table 4) yields another match between the approximated *c*-parameter of 1-docosanol and the *a*- and *b*-parameters of carbon black. This, in theory, should enable the nucleation of the RII phase, disregarding the slightly different lattice parameters of the rotator phase(s). CuO with its monoclinic structure is the best candidate for direct crystal phase nucleation, bypassing the rotator phase(s). C50-OH and C70 are naturally well-suited for nucleation with nearly exact matching of each lattice parameter, respectively, using the same 1,3-distance as before. This may even extend to rotator phases, as C50-OH and C70 potentially exhibit them, too, and their structure can adapt.

**Table 4:** Calculated Lattice Mismatch Parameters for All Face Areas

	ZnO	carbon black	CuO	C50-OH	C70
(a) C22-OH					
a	–63.9%	–72.7%	–48.0%	3.0%	–0.6%
b	–63.9%	–72.7%	–62.0%	–42.6%	–44.8%
c	–42.1%	–25.4%	–43.0%	–72.1%	–72.1%
(b) C22-OH					
a	–34.7%	–50.6%	–6.0%	86.1%	79.7%
b	–34.7%	–50.6%	–31.3%	3.8%	–0.2%
c	4.6%	34.7%	3.0%	–49.5%	–49.5%
(c) C22-OH					
a	29.3%	–2.1%	86.2%	268.7%	256.0%
b	29.3%	–2.1%	36.0%	105.6%	97.7%
c	107.2%	166.9%	104.1%	0.0%^a^	0.0%^a^

a 1,3-approximation.

Ideally, two of the crystal faces match in size, so a set of two lattice parameters of the constituents is similar. This is the case for CuO, but the angle of 99.5° does not match the 1-docosanol angle of 122.5°. Calculating *f* between multiples of each parameter with 1-docosanol provides matches for all materials but requires large defects for nonoverlapping areas. Furthermore, consideration of face areas alone is misleading, as it does not take the shape into account.

The wetting behavior of the nanoparticles was previously tested, showing good compatibility with the aqueous phase. The ZnO and CuO particles do not precipitate or separate visually within 1 day of storage at room temperature. In contrast, carbon black particles separate within a few hours, likely due to their large size, similar to that of the PCS droplets. Unfortunately, none of these nanoparticles with different crystal structures affect supercooling, despite reports in other publications.^43,88^ Although the hexagonal rotator phase matches the crystal structures of ZnO and carbon black, neither shows any impact on the liquid to RII transition. If nucleation were supported by these nanoparticles, it would be expected to manifest here.

The differing crystal structures of the crystalline PCM and the nanoparticles may prevent proper chain alignment and use of the nanoparticle surface as a heterogeneous seed. Although monoclinic CuO is related to the orthorhombic system, differing only in the β angle, it does not yield nucleation at higher temperatures, suggesting that angles should also be considered. Another potential issue is the 1,3-distance assumption for the 1-docosanol c-parameter. Additionally, metal oxide nanoparticles are regularly functionalized with fatty acids, so they could be passivized by surfactant or PCM coordination.^89^ The nucleation support and natural material compatibility of C50-OH with 1-docosanol provide the best results so far, making it suitable for solid phase structure analysis.

### 2.3 Solid Phase Analysis

**Figure 7:**
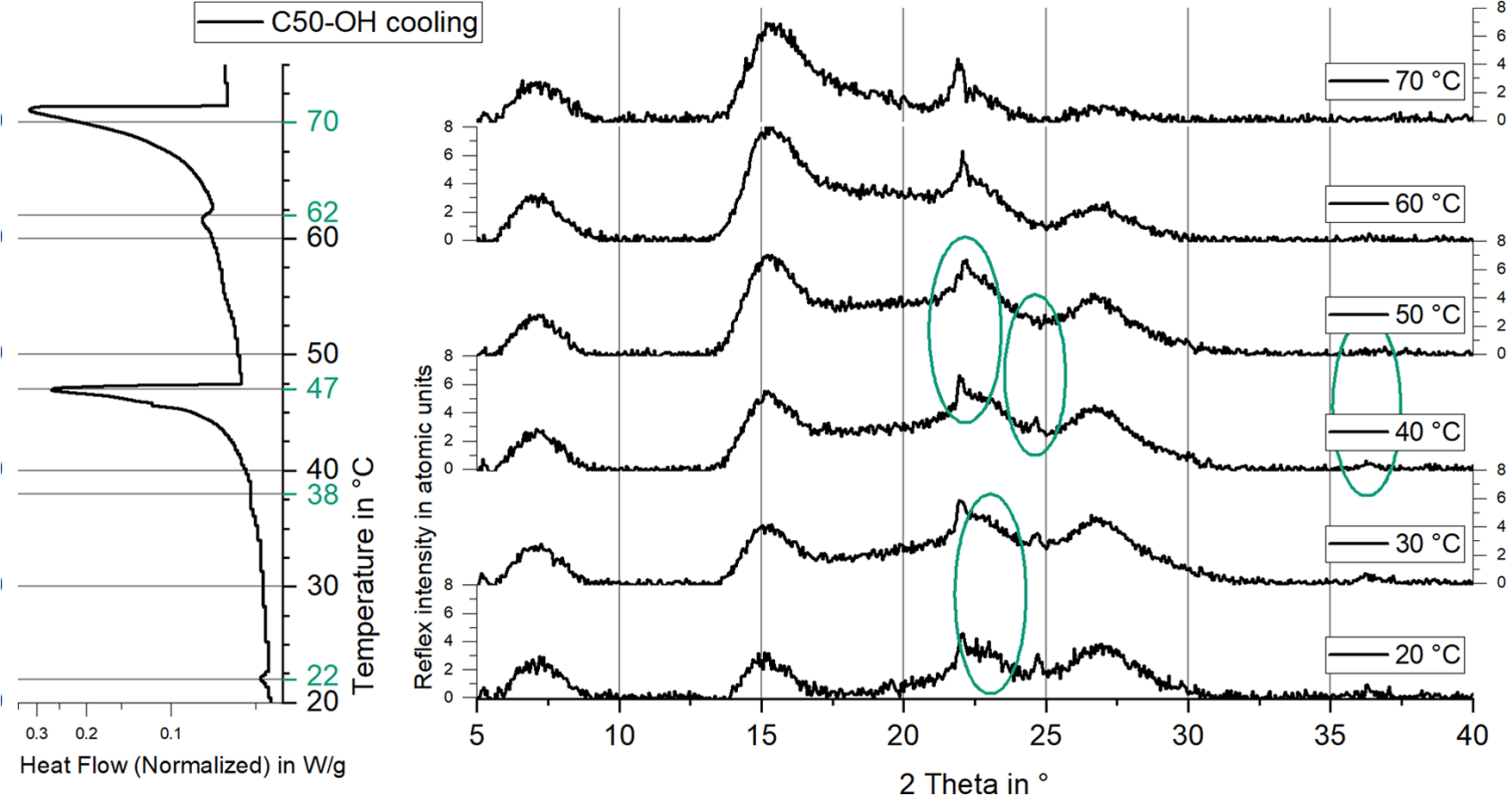
XRD reflexes of a PCS containing 0.8 wt % C50-OH at different temperatures, compared to a DSC enthalpy diagram. The solid phases the PCM is going through in different temperature intervals are visible. These are indicated by changes in the XRD reflex intensity or position (green markup).

The dispersion was analyzed using Cu Kα X-ray diffraction (XRD) in Bragg–Brentano geometry at various temperature steps to observe crystal structure changes during cooling. The results are compared with DSC data to distinguish between multistep crystallization of identical phases and transitions to different crystal phases. Figure 7 correlates the crystallization curve from Figure 5 with the XRD measurement to identify crystal phase transitions at each temperature. Changes in the signal occur after latent heat release in the DSC at specific temperatures. The first XRD signals appear after solidification of the RII phase at 71.7 °C. The transition from RII to RI occurs around 45 °C, indicated by additional reflexes and slight shifts toward smaller angles (expanding structure). Finally, it becomes the orthorhombic crystal near 20 °C, showing additional weak reflexes. This suggests that all transitions in these areas belong to one crystal phase, with colder transitions resulting from delayed nucleation. A limitation of 20 wt % PCM emulsion XRD measurements is the low signal-to-noise ratio, even at long exposure times, complicating the identification of PCM crystal peaks, which can be overshadowed by background signals. Since the particle size distribution does not indicate large particles, a working seed should be present in almost all particles, especially as the second DSC peak shifts nearly completely. Only the small, delayed peak from the RII phase at 62.9 °C, containing about 1% enthalpy, remains.

**Figure 8:**
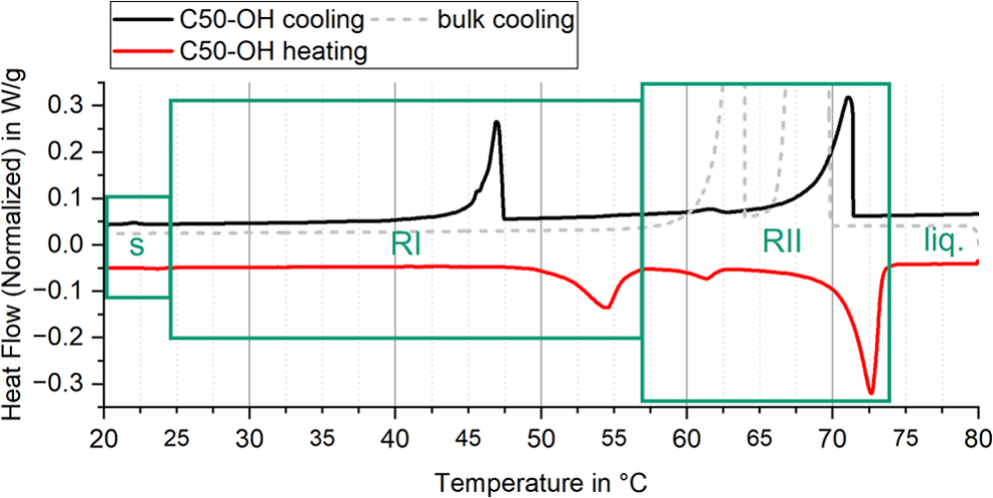
Assignment of the different melting and freezing transitions to separate phases solid, RI, RII (green boxes, left to right) and liquid.

Assuming that the corresponding transitions on the heating curve represent the stable temperatures of these phases, the transitions in Figure 8 can be distinguished as individual events based on the XRD results. If the solid phases do not influence each other and can be approximated separately, the remaining supercooling shifting potential lies in the leftover enthalpy of the small transition liquid to RII at 62.5 °C and in the RII to RI transition at 47.4 °C. Shifting all transitions to the highest melting onset (70.7 °C) offers greater potential, but no evidence has been found to support this possibility so far.

### 2.4 Seed Deactivation

**Figure 9:**
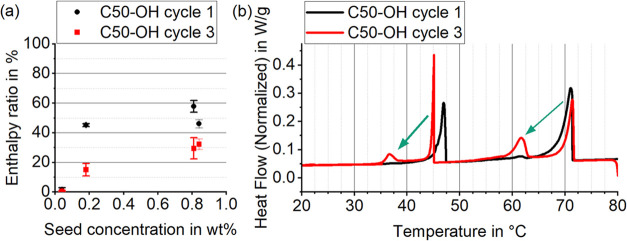
(a) Decrease in nucleation ratio over increasing number of thermal cycles, observed in PCS with seed C50-OH. (b) The enthalpy shifts back to (higher) supercooling.

The paraffin nucleation additives C50-OH (Figure 9a) and C70 (Figure S2) exhibit a steadily declining ER with an increasing number of thermal cycles. Specifically, the transitions initially shifted to higher temperatures gradually return to lower temperatures with each cycle (Figure 9b). The XRD results show no changes within the shifting regions Tc1–Tc2 and Tc3–Tc4, supporting the assumption that these transitions belong to the same crystal phase but have different nucleation temperatures.

The thermal history of the dispersions can be reset by heating them above the melting point of the seed material, restoring the seed’s effect. However, deactivation recurs with further cycling. The exact mechanism is unclear, but passivation or separation of the seed, potentially by surfactants, is suggested. This process must occur within the droplet, as the behavior is reversible. Internal separation may result from buoyancy forces due to the density difference between the solid seed and the liquid PCM.^90^ Once the seed moves to the droplet interface, it can be passivized by surfactants covering its surface, rendering it unavailable for nucleation in the PCM. Notably, the ER decrease is less pronounced in samples with higher seed concentrations, as more seed must be passivized before losing functionality.

### 2.5 Surfactant Impact

**Figure 10:**
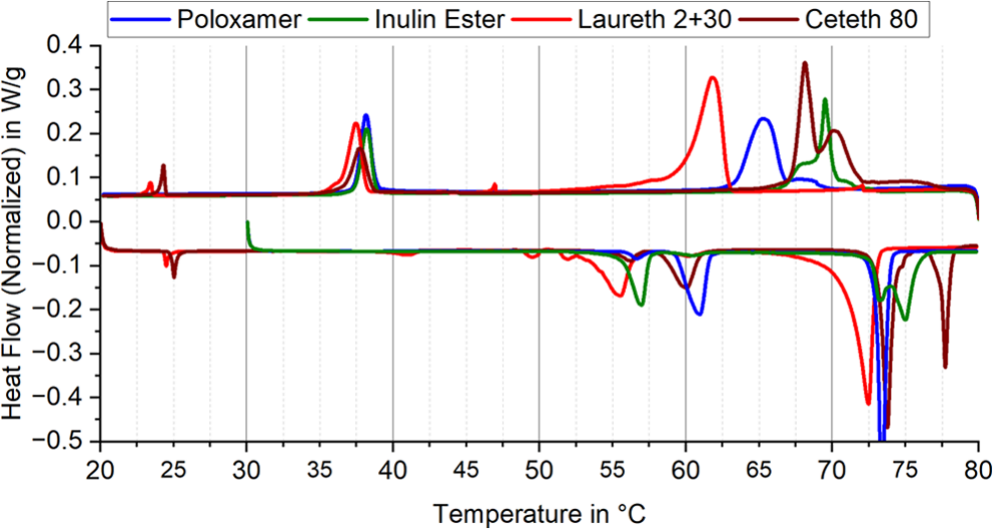
Comparison of DSC heat flow diagrams of 1-docosanol dispersions with different surfactants, but without seed.

Different surfactants show different degrees of supercooling in C22-OH dispersions. For fatty alcohols, an optimal HLB value is around 16 with linear surfactants. Figure 10 compares the heat flow diagrams of dispersions with similar particle sizes (Figure S3) using different surfactants. All dispersions have a d90 below 1 μm, with only the d50 of the dispersion with Laureth-2 and Laureth-30 slightly higher than the others. Crystallization is significantly influenced by the surfactant, while the low-temperature phase transition (around 38 °C) remains consistent across dispersions. A similar behavior was observed in alkane nano emulsions at different temperatures.^38,91,92^ Emulsions with Inulin-Ester and Ceteth-80 show the highest crystallization temperatures, followed by Poloxamer, and finally the Laureth mixture with the lowest transition temperature. Interestingly, the melting temperatures shift in the same order but to slightly higher temperatures. This phenomenon is attributed to the intercalation of water molecules in the fatty alcohol structure during heating, stabilizing it through additional hydrogen bonding between functional groups.^93^ In bulk material, this is not observed due to slow water diffusion compared to the large volume.

The melting curves in Figure 10 display similar peaks at higher temperatures with the Laureth-mixture PCS onset occurring slightly below. The emulsions with Inulin-Ester and Ceteth-80 show a two-step phase transition, reflected in the crystallization curves, possibly due to a bimodal particle size distribution. The lower temperature solid–solid transitions occur slightly offset during heating, indicating that both crystallization and melting processes are influenced by component composition and are interrelated. Contrary to the findings of Dungan et al., both droplet size and emulsifier type significantly impact droplet melting behavior. However, experiments consistently show that emulsifiers with hydrocarbon tails similar to the PCM facilitate crystallization at higher temperatures.^51^ Günther et al. and Huang et al. conducted experimental studies on paraffin-in-water emulsions and similarly observed that surfactants significantly influenced supercooling phenomena. The smaller the droplets, the greater the supercooling, and different surfactants had varying effects on melting and solidification temperatures.^85,94^ Ceteth-80 demonstrates particularly low supercooling, likely because its lipophilic part (18 carbon atoms) resembles the PCM more than Laureth (12 carbon atoms).^95^ Inulin, esterified with Lauric acid, differs from Laureth due to its polymeric backbone, which is entirely hydrophilic and theoretically does not contact the PCM directly. The distance between internal lauryl groups in inulin may allow PCM seeds to form and trigger crystallization, unlike tightly packed ethoxylates Laureth and Ceteth.^85^ Poloxamer, a block copolymer with hydrophilic and hydrophobic blocks, seems to form a structure partly compatible with the PCM while the other parts do not inhibit nucleation. Ultimately, different surfactants affect the position of the first supercooled peak, primarily influencing the liquid to RII transition like the seeds, with minimal changes at other peaks (±2 K).

## 3 Conclusions

Supercooling in organic phase change material emulsions (or slurries, “PCS”), specifically those containing 1-docosanol (C22-OH), poses a significant challenge for thermal energy storage applications by reducing efficiency and reliability. This phenomenon is particularly pronounced in small particles due to increased nucleation barriers that delay crystallization. This study demonstrates that supercooling in oil-in-water PCS can be mitigated effectively using nucleating agents that share structural similarities with the phase change material (PCM), particularly during the initial cooling cycle. Rotor-stator emulsification techniques successfully produce stable PCS with particle size distributions below 1 μm that show increased supercooling. The addition of higher paraffins, such as C50-OH and C70, as nucleation agents in concentrations below 1 wt % significantly reduces supercooling. Differential scanning calorimetry (DSC) analysis reveals that these nucleating agents effectively shift most of the latent heat release to higher temperatures closer to the PCM’s melting point. In this study, the maximum nucleation ratio without significant supercooling is approximately 60% of the PCM’s latent heat within a 10 K temperature interval. While the results indicate that C70 may have better nucleating properties, C50-OH proves to be more reliable and is thus recommended for use with C22-OH. The liquid-to-rotator phase 2 (RII) transition exhibits the most potential for supercooling reduction, while the influence on solid–solid transitions varies. X-ray diffraction (XRD) measurements further confirm the structural phase transitions observed in the DSC analysis. They demonstrate that the liquid-to-RII transition occurs at higher temperatures when nucleating agents are present, indicating a successful alteration of the crystallization pathway. Although all supercooled phases can be shifted toward their thermodynamic transition temperatures, fully eliminating supercooling remains challenging due to thermodynamic limits. The effectiveness of nucleation agents is attributed to the high structural compatibility between the PCM and the seed material. Seeds with low nucleation barriers due to structural similarity decrease supercooling effectively. Surfactants also influence nucleation; certain surfactant structures either complement the other components to support nucleation within the droplets or, if incompatible, may hinder crystallization by interfering with nucleation sites.

Future research should focus on a deeper understanding of the mechanisms underlying supercooling in PCMs. Exploring additional nucleating agents, such as nanoparticles with exact crystal lattice matching to the PCM, may provide new ways for supercooling mitigation. Functionalization of these materials could enhance their compatibility and effectiveness as nucleating agents. Molecular dynamics simulations are recommended to provide detailed insights into the nucleation processes occurring within the droplets. Such simulations can elucidate the exact mechanisms by which nucleating agents influence crystallization, aiding in the design of more effective strategies to reduce supercooling. Addressing the issue of seed deactivation over multiple thermal cycles is essential for the long-term reliability of PCS applications. Understanding the factors leading to seed deactivation, such as surfactant interference or separation within droplets, will be critical in developing strategies to maintain the effectiveness of nucleating agents. By carefully selecting and optimizing all components of a PCS it is possible to minimize supercooling and maximize the latent heat storage capacity. Advancements in this area will enhance the performance of thermal energy storage systems, e.g., in better peak load buffering, and applications in systems where space is limited. Ultimately, this contributes to a potential increase in the efficiency of thermal energy systems and supports the development of more effective energy storage solutions.

## 4 Experimental Section

All chemicals were used as received, without further purification. The phase change material (PCM) used was 1-docosanol (behenyl alcohol, C22-OH), supplied by Rubitherm. This PCM has a melting point of approximately 70 °C and a purity of approximately 97%, containing minor fractions of fatty alcohols with slightly different chain lengths. Details on surfactants (Table S1) and nucleation additives (Table S2) are given in the Supporting Information. Deionized water used for the emulsions was taken from the in-house reverse osmosis purification system. Both the dispersed and continuous phases of the phase change slurry (PCS) were prepared separately. The desired surfactants were completely dissolved at temperatures above the PCM melting point to ensure full solubility. The dispersed phase was then added to the continuous phase under rapid stirring (>500 rpm) to create a pre-emulsion. After removing the magnetic stirrer, the emulsion was processed using a dispersing device - either a rotor-stator mixer (IKA MagicLab) operated for 10 min at 18000 rpm or an ultrasonic probe (Branson SFX 550, generator model 902R) for 3 min at 275 W. Subsequently, the emulsions were homogenized using a two-step high-pressure homogenizer (APV model) at pressures of 200/20 bar for two cycles. All equipment was kept at temperatures above the PCM melting point during processing to prevent premature solidification. Soluble nucleating additives were added directly to the PCM at elevated temperatures to ensure complete dissolution and mixing. Insoluble additives were dispersed within the PCM before combining the oil and water phases. After the addition of nucleating additives, the emulsions were prepared following the same procedure described above.

Thermal characterization was performed using differential scanning calorimetry (DSC) (TA Instruments, Discovery DSC 2500). Samples were heated and cooled between 20 and 80 °C at a rate of 1 K/min, with isothermal periods between the heating and cooling ramps to ensure thermal equilibrium. Sealed aluminum crucibles were utilized to prevent sample drying and maintain consistent sample mass. Typical DSC samples contained 10 to 20 mg of sample. A minimum of three thermal cycles were conducted to identify any measurement irregularities; additional cycles were performed if necessary to ensure reproducibility. The device was temperature and enthalpy calibrated using Ga, In, Bi, Sn and Pb standards (PTB certified) during melting, covering heating rates from 0.25 to 10 K/min. The deviation of onset temperatures was within 0.1 K and the deviation of enthalpy values was within 2% of literature values of the calibration samples. Nitrogen gas (6.0) was used for purging at 50 mL/min. The melting enthalpy error values include 2% device uncertainty and the repeated measurements standard deviation, added up by linear error propagation.

Particle size distributions were measured using static light scattering (SLS) with a Beckman Coulter LS 13 320 instrument equipped with a Universal Liquid Module (ULM). Mie scattering theory was applied using the Polarization Intensity Differential Scattering (PIDS) module, with a real refractive index of 1.46 and an imaginary refractive index of 0.005 for the dispersed phase. Samples were diluted to a measurement concentration of 2% to prevent multiple scattering effects. Intensity data were collected over 90 s for each of three repeated measurements, and the results were averaged to obtain the final particle size distribution.

Structural analysis of the materials was conducted using X-ray diffraction (XRD) with a Bruker D8 Advance diffractometer in Bragg–Brentano geometry. Instrument settings included a primary radius of 260 mm, a secondary radius of 300 mm, Cu Kα radiation (λ = 1.5406 Å), Soller slits with an angle of 4.1°, and a fixed divergence slit size of 6 mm. A position-sensitive detector was utilized for data collection. XRD measurements were performed at various temperature steps using a heating stage to observe phase transitions. Samples were placed in a metal chamber and sealed with Kapton tape to prevent water evaporation during heating. Reference signals from the sample holder and Kapton tape were subtracted during data analysis.

## Supporting Information Available

Additional material properties and emulsion formulations are provided in tables (Table S1: surfactants, Table S2: nucleation additives, Table S3: emulsion formulations). Figures S1 and S3 show particle size distributions and Figure S2 shows seed deactivation with C70 (PDF)

## Nomenclature

PCM: phase change material
PCS: phase change slurry
O/W: oil-in-water
ER: enthalpy ratio
XRD: X-ray diffraction
DSC: differential scanning calorimetry
HLB: hydrophilic–lipophilic balance
RI: rotator phase 1
RII: rotator phase 2
Tc: temperature of transition

## References

[ref1] Cabaleiro D.; Agresti F.; Fedele L.; Barison S.; Hermida-Merino C.; Losada-Barreiro S.; Bobbo S.; Piñeiro M. M. Review on phase change material emulsions for advanced thermal management: Design, characterization and thermal performance. Renewable Sustainable Energy Rev. 2022, 159, 112238 10.1016/j.rser.2022.112238.

[ref2] Dash L.; Anna Mahanwar P. A review on organic phase change materials and their applications. Int. J. Eng. Appl. Sci. Technol. 2021, 5, 268–284. 10.33564/IJEAST.2021.v05i09.045.

[ref3] Jankowski N. R.; McCluskey F. P. A review of phase change materials for vehicle component thermal buffering. Applied Energy 2014, 113, 1525–1561. 10.1016/j.apenergy.2013.08.026.

[ref4] Sharma A.; Chauhan R.; Ali Kallioğlu M.; Chinnasamy V.; Singh T. A review of phase change materials (PCMs) for thermal storage in solar air heating systems. Mater. Today: Proc. 2021, 44, 4357–4363. 10.1016/j.matpr.2020.10.560.

[ref5] Safari A.; Saidur R.; Sulaiman F. A.; Xu Y.; Dong J. A review on supercooling of Phase Change Materials in thermal energy storage systems. Renewable Sustainable Energy Rev. 2017, 70, 905–919. 10.1016/j.rser.2016.11.272.

[ref6] Shamseddine I.; Pennec F.; Biwole P.; Fardoun F. Supercooling of phase change materials: A review. Renewable Sustainable Energy Rev. 2022, 158, 112172 10.1016/j.rser.2022.112172.

[ref7] Truong T.; Bansal N.; Sharma R.; Palmer M.; Bhandari B. Effects of emulsion droplet sizes on the crystallisation of milk fat. Food Chem. 2014, 145, 725–735. 10.1016/j.foodchem.2013.08.072. 24128537

[ref8] Han J.-W.; Zuo M.; Zhu W.-Y.; Zuo J.-H.; Lü E.-L.; Yang X.-T. A comprehensive review of cold chain logistics for fresh agricultural products: Current status, challenges, and future trends. Trends Food Sci. Technol. 2021, 109, 536–551. 10.1016/j.tifs.2021.01.066.

[ref9] Ahmed J.; Taher A.; Mulla M. Z.; Al-Hazza A.; Luciano G. Effect of sieve particle size on functional, thermal, rheological and pasting properties of Indian and Turkish lentil flour. J. Food Eng. 2016, 186, 34–41. 10.1016/j.jfoodeng.2016.04.008.

[ref10] ACS Symp. Ser.; Zuidam N. J.; Nedovic V., Eds.; Springer New York: New York, NY, 2010; Vol. 1331.

[ref11] Food Emulsions and Foams; Elsevier, 2005.

[ref12] Yang Y.; Marshall-Breton C.; Leser M. E.; Sher A. A.; McClements D. J. Fabrication of ultrafine edible emulsions: Comparison of high-energy and low-energy homogenization methods. Food Hydrocolloids 2012, 29, 398–406. 10.1016/j.foodhyd.2012.04.009.

[ref13] Drago E.; Campardelli R.; Pettinato M.; Perego P. Innovations in Smart Packaging Concepts for Food: An Extensive Review. Foods 2020, 9, 1628 10.3390/foods9111628. 33171881 PMC7695158

[ref14] Lin W.; Gschwander S.; Song W.; Feng Z.; Farid M. M. Preparation, characterisation and property modification of a calcium chloride hexahydrate phase change material slurry with additives for thermal energy transportation. International Journal of Refrigeration 2024, 160, 312–328. 10.1016/j.ijrefrig.2024.02.010.

[ref15] Chen J.; Zhang P. Preparation and characterization of nano-sized phase change emulsions as thermal energy storage and transport media. Applied Energy 2017, 190, 868–879. 10.1016/j.apenergy.2017.01.012.

[ref16] IEEE, An Evaluation Of Microencapsulated PCM For Use In Cold Energy Transportation Medium - Energy Conversion Engineering Conference, 1996. IECEC 96., Proceedings of the 31st Intersociety.

[ref17] Rakkappan S. R.; Sivan S.; Ahmed S. N.; Naarendharan M.; Sai Sudhir P. Preparation, characterisation and energy storage performance study on 1-Decanol-Expanded graphite composite PCM for air-conditioning cold storage system. Int. J. Refrigeration 2021, 123, 91–101. 10.1016/j.ijrefrig.2020.11.004.

[ref18] Nie B.; Du Z.; Chen J.; Zou B.; Ding Y. Performance enhancement of cold energy storage using phase change materials with fumed silica for air-conditioning applications. Int. J. Energy Res. 2021, 45, 16565–16575. 10.1002/er.6903.

[ref19] L.T.D. Inc., A phase change materials slurry to decrease peak air conditioning loads, in C.E. Commission (Ed.) CEC-500-2006-026, http://400.sydneyplus.com/CaliforniaEnergy_SydneyEnterprise/Portal/public.aspx?component=AAAAIY&record=aed0d78a-8349-4d19-9722-570c8e26cc3f.

[ref20] Li G.; Hwang Y.; Radermacher R. Review of cold storage materials for air conditioning application. Int. J. Refrigerat. 2012, 35, 2053–2077. 10.1016/j.ijrefrig.2012.06.003.

[ref21] McClements D. J. Crystals and crystallization in oil-in-water emulsions: implications for emulsion-based delivery systems. Adv. Colloid Interface Sci. 2012, 174, 1–30. 10.1016/j.cis.2012.03.002. 22475330

[ref22] Lane G. A. Phase change materials for energy storage nucleation to prevent supercooling. Sol. Energy Mater. Sol. Cells 1992, 27, 135–160. 10.1016/0927-0248(92)90116-7.

[ref23] Pathak L.; Trivedi G.; Parameshwaran R.; Deshmukh S. S. Microencapsulated phase change materials as slurries for thermal energy storage: A review. Mater. Today: Proc. 2021, 44, 1960–1963. 10.1016/j.matpr.2020.12.101.

[ref24] Delgado M.; Lázaro A.; Mazo J.; Zalba B. Review on phase change material emulsions and microencapsulated phase change material slurries: Materials, heat transfer studies and applications. Renewable Sustainable Energy Rev. 2012, 16, 253–273. 10.1016/j.rser.2011.07.152.

[ref25] Shao J.; Darkwa J.; Kokogiannakis G. Review of phase change emulsions (PCMEs) and their applications in HVAC systems. Energy Buildings 2015, 94, 200–217. 10.1016/j.enbuild.2015.03.003.

[ref26] Wang F.; Lin W.; Ling Z.; Fang X. A comprehensive review on phase change material emulsions: Fabrication, characteristics, and heat transfer performance. Sol. Energy Mater. Sol. Cells 2019, 191, 218–234. 10.1016/j.solmat.2018.11.016.

[ref27] Sirota E. B. Supercooling, Nucleation, Rotator Phases, and Surface Crystallization of n -Alkane Melts. Langmuir 1998, 14, 3133–3136. 10.1021/la970594s.

[ref28] Abramov S.; Shah K.; Weißenstein L.; Karbstein H. Effect of Alkane Chain Length on Crystallization in Emulsions during Supercooling in Quiescent Systems and under Mechanical Stress. Processes 2018, 6, 6 10.3390/pr6010006.

[ref29] Zhang X.-x.; Fan Y.; Tao X.; Yick K. Crystallization and prevention of supercooling of microencapsulated n-alkanes. J. Colloid Interface Sci. 2005, 281, 299–306. 10.1016/j.jcis.2004.08.046. 15571685

[ref30] Noël J. A.; Kreplak L.; Getangama N. N.; de Bruyn J. R.; White M. A. Supercooling and Nucleation of Fatty Acids: Influence of Thermal History on the Behavior of the Liquid Phase. J. Phys. Chem. B 2018, 122, 12386–12395. 10.1021/acs.jpcb.8b10568. 30507193

[ref31] Royon L.; Guiffant G. Heat transfer in paraffin oil/water emulsion involving supercooling phenomenon. Energy Convers. Manage. 2001, 42, 2155–2161. 10.1016/S0196-8904(00)00130-8.

[ref32] Hagelstein G.; Gschwander S. Reduction of supercooling in paraffin phase change slurry by polyvinyl alcohol. Int. J. Refrigerat. 2017, 84, 67–75. 10.1016/j.ijrefrig.2017.08.016.

[ref33] Xiang N.; Yuan Y.; Sun L.; Cao X.; Zhao J. Simultaneous decrease in supercooling and enhancement of thermal conductivity of paraffin emulsion in medium temperature range with graphene as additive. Thermochim. Acta 2018, 664, 16–25. 10.1016/j.tca.2018.04.004.

[ref34] Montenegro R.; Antonietti M.; Mastai Y.; Landfester K. Crystallization in Miniemulsion Droplets. J. Phys. Chem. B 2003, 107, 5088–5094. 10.1021/jp0262057.

[ref35] Douaire M.; Di Bari V.; Norton J. E.; Sullo A.; Lillford P.; Norton I. T. Fat crystallisation at oil-water interfaces. Adv. Colloid Interface Sci. 2014, 203, 1–10. 10.1016/j.cis.2013.10.022. 24238924

[ref36] Smit W. J.; Smolentsev N.; Versluis J.; Roke S.; Bakker H. J. Freezing effects of oil-in-water emulsions studied by sum-frequency scattering spectroscopy. J. Chem. Phys. 2016, 145, 44706 10.1063/1.4959128. 27475385

[ref37] Mura E.; Ding Y. Nucleation of melt: From fundamentals to dispersed systems. Adv. Colloid Interface Sci. 2021, 289, 102361 10.1016/j.cis.2021.102361. 33561567

[ref38] Hagelstein G. Untersuchung zum Kristallisationsverhalten in n-Octadecan-Wasser-Dispersionen. INAUGURALDISSERTATION, 2018.

[ref39] Morimoto T.; Kawana Y.; Saegusa K.; Kumano H. Supercooling characteristics of phase change material particles within phase change emulsions. Int. J. Refrigerat. 2019, 99, 1–7. 10.1016/j.ijrefrig.2018.11.039.

[ref40] El Rhafiki T.; Kousksou T.; Jamil A.; Jegadheeswaran S.; Pohekar S. D.; Zeraouli Y. Crystallization of PCMs inside an emulsion: Supercooling phenomenon. Sol. Energy Mater. Sol. Cells 2011, 95, 2588–2597. 10.1016/j.solmat.2011.03.027.

[ref41] Gschwander S.; Niedermaier S.; Gamisch S.; Kick M.; Klünder F.; Haussmann T. Storage Capacity in Dependency of Supercooling and Cycle Stability of Different PCM Emulsions. Appl. Sci. 2021, 11, 3612 10.3390/app11083612.

[ref42] Liu L.; Niu J.; Wu J.-Y. Formulation of highly stable PCM nano-emulsions with reduced supercooling for thermal energy storage using surfactant mixtures. Sol. Energy Mater. Sol. Cells 2021, 223, 110983 10.1016/j.solmat.2021.110983.

[ref43] Wang F.; Zhang C.; Liu J.; Fang X.; Zhang Z. Highly stable graphite nanoparticle-dispersed phase change emulsions with little supercooling and high thermal conductivity for cold energy storage. Applied Energy 2017, 188, 97–106. 10.1016/j.apenergy.2016.11.122.

[ref44] Wang F.; Fang X.; Zhang Z. Preparation of phase change material emulsions with good stability and little supercooling by using a mixed polymeric emulsifier for thermal energy storage. Sol. Energy Mater. Sol. Cells 2018, 176, 381–390. 10.1016/j.solmat.2017.10.025.

[ref45] Xie B.; Liu G.; Jiang S.; Zhao Y.; Wang D. Crystallization behaviors of n-octadecane in confined space: crossover of rotator phase from transient to metastable induced by surface freezing. J. Phys. Chem. B 2008, 112, 13310–13315. 10.1021/jp712160k. 18816090

[ref46] Coupland J. N., Crystallization of Lipids in Oil-in-Water Emulsion States, 431–446, DOI 10.1002/9781118593882.ch15.

[ref47] Dickinson E.; Kruizenga F.-J.; Povey M. J.; van der Molen M. Crystallization in oil-in-water emulsions containing liquid and solid droplets. Colloids Surf., A 1993, 81, 273–279. 10.1016/0927-7757(93)80255-D.

[ref48] Hindle S.; Povey M. J.; Smith K. Kinetics of Crystallization in n-Hexadecane and Cocoa Butter Oil-in-Water Emulsions Accounting for Droplet Collision-Mediated Nucleation. J. Colloid Interface Sci. 2000, 232, 370–380. 10.1006/jcis.2000.7174. 11097773

[ref49] Gülseren İ.; Coupland J. N. Surface Melting in Alkane Emulsion Droplets as Affected by Surfactant Type. J. Am. Oil Chem. Soc. 2008, 85, 413–419. 10.1007/s11746-008-1216-z.

[ref50] Gülseren İ.; Coupland J. N. The Effect of Emulsifier Type and Droplet Size on Phase Transitions in Emulsified Even-Numbered n -Alkanes. J. Am. Oil Chem. Soc. 2007, 84, 621–629. 10.1007/s11746-007-1093-x.

[ref51] McClements D. J.; Dungan S. R.; German J. B.; Simoneau C.; Kinsella J. E. Droplet Size and Emulsifier Type Affect Crystallization and Melting of Hydrocarbon-in-Water Emulsions. J. Food Sci. 1993, 58, 1148–1151. 10.1111/j.1365-2621.1993.tb06135.x.

[ref52] Garti N.; Wellner E.; Sarig S. Effect of surfactants on crystal structure modification of stearic acid. J. Cryst. Growth 1982, 57, 577–584. 10.1016/0022-0248(82)90076-8.

[ref53] Golemanov K.; Tcholakova S.; Denkov N. D.; Gurkov T. Selection of surfactants for stable paraffin-in-water dispersions, undergoing solid-liquid transition of the dispersed particles. Langmuir 2006, 22, 3560–3569. 10.1021/la053059y. 16584227

[ref54] Zhang X.; Niu J.; Zhang S.; Wu J.-Y. PCM in Water Emulsions: Supercooling Reduction Effects of Nano-Additives, Viscosity Effects of Surfactants and Stability. Adv. Eng. Mater. 2015, 17, 181–188. 10.1002/adem.201300575.

[ref55] Ueno S.; Hamada Y.; Sato K. Controlling Polymorphic Crystallization of n -Alkane Crystals in Emulsion Droplets through Interfacial Heterogeneous Nucleation. Cryst. Growth Des. 2003, 3, 935–939. 10.1021/cg0300230.

[ref56] Kaneko F.; Yamamoto Y.; Yoshikawa S. Structural Study on Fat Crystallization Process Heterogeneously Induced by Graphite Surfaces. Molecules 2020, 25, 4786 10.3390/molecules25204786. 33086514 PMC7587562

[ref57] Arima S.; Ueno S.; Ogawa A.; Sato K. Scanning microbeam small-angle X-ray diffraction study of interfacial heterogeneous crystallization of fat crystals in oil-in-water emulsion droplets. Langmuir 2009, 25, 9777–9784. 10.1021/la901115x. 19588887

[ref58] Phipps L. W. Heterogeneous and homogeneous nucleation in supercooled triglycerides and n-paraffins. Trans. Faraday Soc. 1964, 60, 1873 10.1039/tf9646001873.

[ref59] Shinohara Y.; Takamizawa T.; Ueno S.; Sato K.; Kobayashi I.; Nakajima M.; Amemiya Y. Microbeam X-ray Diffraction Analysis of Interfacial Heterogeneous Nucleation of n -Hexadecane inside Oil-in-Water Emulsion Droplets. Cryst. Growth Des. 2008, 8, 3123–3126. 10.1021/cg701018x.

[ref60] Mukherjee P. K. Effect of the liquid crystal solute on the rotator phase transitions of n-alkanes. RSC Adv. 2015, 5, 12168–12177. 10.1039/C4RA14116D.

[ref61] Chazhengina S.; Kotelnikova E.; Filippova I.; Filatov S. Phase transitions of n-alkanes as rotator crystals. J. Mol. Struct. 2003, 647, 243–257. 10.1016/S0022-2860(02)00531-8.

[ref62] Denicolò I.; Doucet J.; Craievich A. F. X-ray study of the rotator phase of paraffins (III): Even-numbered paraffins C18H38, C2H42, C22H46, C24H5, and C26H54. J. Chem. Phys. 1983, 78, 1465–1469. 10.1063/1.444835.

[ref63] Dirand M.; Achour Z.; Jouti B.; Sabour A.; Gachon J.-C. Binary Mixtures of n-Alkanes. Phase Diagram Generalization: Intermediate Solid Solutions, Rotator Phases. Mol. Cryst. Liq. Cryst. Sci. Technol., Sect. A 1996, 275, 293–304. 10.1080/10587259608034082.

[ref64] Mukherjee P. K. Phase transitions among the rotator phases of the normal alkanes: A review. Phys. Rep. 2015, 588, 1–54. 10.1016/j.physrep.2015.05.005.

[ref65] Doucet J.; Denicolo I.; Craievich A. X-ray study of the ‘rotator’’ phase of the odd-numbered paraffins C17H36, C19H40, and C21H44. J. Chem. Phys. 1981, 75, 1523–1529. 10.1063/1.442185.

[ref66] Doucet J.; Denicolò I.; Craievich A. F.; Germain C. X-ray study of the rotator phase of paraffins (IV): C27H56, C28H58, C29H6, C3H62, C32H66, and C34H7. J. Chem. Phys. 1984, 80, 1647–1651. 10.1063/1.446865.

[ref67] Sirota E. B.; Herhold A. B. Transient Rotator Phase Induced Nucleation in n -Alkanes. In ACS Symposium Series pp 232–241.

[ref68] Nozaki K.; Hikosaka M. Mechanism of primary nucleation and origin of hysteresis in the rotator phase transition of an Odd n-alkane. J. Mater. Sci. 2000, 35, 1239–1252. 10.1023/A:1004752907345.

[ref69] Sirota E. B.; Singer D. M. Phase transitions among the rotator phases of the normal alkanes. J. Chem. Phys. 1994, 101, 10873–10882. 10.1063/1.467837.

[ref70] Shinohara Y.; Kawasaki N.; Ueno S.; Kobayashi I.; Nakajima M.; Amemiya Y. Observation of the transient rotator phase of n-hexadecane in emulsified droplets with time-resolved two-dimensional small- and wide-angle X-ray scattering. Phys. Rev. Lett. 2005, 94, 97801 10.1103/PhysRevLett.94.097801. 15784000

[ref71] Sirota E. B.; King H. E.; Singer D. M.; Shao H. H. Rotator phases of the normal alkanes: An x-ray scattering study. J. Chem. Phys. 1993, 98, 5809–5824. 10.1063/1.464874.

[ref72] Zahir M. H.; Mohamed S. A.; Saidur R.; Al-Sulaiman F. A. Supercooling of phase-change materials and the techniques used to mitigate the phenomenon. Appl. Energy 2019, 240, 793–817. 10.1016/j.apenergy.2019.02.045.

[ref73] Tafelmeier S.; Hiebler S. Molecular Dynamics Simulation of the Crystallization Behavior of Octadecane on a Homogeneous Nucleus. Crystals 2022, 12, 987 10.3390/cryst12070987.

[ref74] Iliev S.; Tsibranska S.; Ivanova A.; Tcholakova S.; Denkov N. Computational assessment of hexadecane freezing by equilibrium atomistic molecular dynamics simulations. J. Colloid Interface Sci. 2023, 638, 743–757. 10.1016/j.jcis.2023.01.126. 36780853

[ref75] Kick M.; Gschwander S., Reduction of rotation phase supercooling in n-docosanol nano phase change slurries 10.18462/IIR.PCM.2021.2042.

[ref76] Leunissen M. E.; Graswinckel W. S.; van Enckevort W. J. P.; Vlieg E. Epitaxial Nucleation and Growth of n -Alkane Crystals on Graphite (0001). Cryst. Growth Des. 2004, 4, 361–367. 10.1021/cg0340852.

[ref77] Oxtoby D. W. Homogeneous nucleation: theory and experiment. J. Chem. Phys. 1992, 4, 7627–7650. 10.1088/0953-8984/4/38/001.

[ref78] Griffin W. C. Classification of surface active agents by HLB. J. Society Cosmetic Chem. 1946, 311–326.

[ref79] Delgado M.; Lázaro A.; Peñalosa C.; Mazo J.; Zalba B. Analysis of the physical stability of PCM slurries. Int. J. Refrigerat. 2013, 36, 1648–1656. 10.1016/j.ijrefrig.2013.04.020.

[ref80] Yamashita Y.; Miyahara R.; Sakamoto K. Cosmetic Science and Technology; Elsevier, 2017; pp 489–506.

[ref81] H.-D., Dörfler Grenzflächen- und Kolloidchemie; VCH, Weinheim, 1994.

[ref82] Sirota E. B.; Wu X. Z. The rotator phases of neat and hydrated 1-alcohols. J. Chem. Phys. 1996, 105, 7763–7773. 10.1063/1.472559.

[ref83] Tanaka K.; Seto T.; Hayashida T. Phase Transformation of n-Higher Alcohols. (I). Bulletin of the Institute for Chemical Research; Kyoto University, 1958; pp 123–139.

[ref84] Kaneko N.; Horie T.; Ueno S.; Yano J.; Katsuragi T.; Sato K. Impurity effects on crystallization rates of n-hexadecane in oil-in-water emulsions. J. Cryst. Growth 1999, 197, 263–270. 10.1016/S0022-0248(98)00933-6.

[ref85] Günther E.; Huang L.; Mehling H.; Dötsch C. Subcooling in PCM emulsions – Part 2: Interpretation in terms of nucleation theory. Thermochim. Acta 2011, 522, 199–204. 10.1016/j.tca.2011.04.027.

[ref86] Precht D. Kristallstrukturuntersuchungen an Fettalkoholen und Fettsäuren mit Elektronen- und Röntgenbeugung I. Fette, Seifen, Anstrichm. 1976, 78, 145–149. 10.1002/lipi.19760780402.

[ref87] Precht D. Kristallstrukturuntersuchungen an Fettalkoholen und Fettsäuren mit Elektronen- und Röntgenbeugung II. Fette, Seifen, Anstrichm. 1976, 78, 189–192. 10.1002/lipi.19760780503.

[ref88] Tang J.; Wang Y.; Liu H.; Belfiore L. A. Effects of organic nucleating agents and zinc oxide nanoparticles on isotactic polypropylene crystallization. Polymer 2004, 45, 2081–2091. 10.1016/j.polymer.2003.11.046.

[ref89] Li C.-C.; Chang M.-H. Colloidal stability of CuO nanoparticles in alkanes via oleate modifications. Mater. Lett. 2004, 58, 3903–3907. 10.1016/j.matlet.2004.05.088.

[ref90] Piazza R.; Buzzaccaro S.; Secchi E.; Parola A. On the general concept of buoyancy in sedimentation and ultracentrifugation. Phys. Biol. 2013, 10, 45005 10.1088/1478-3975/10/4/045005. 23913160

[ref91] Turnbull D.; Cormia R. L. Kinetics of Crystal Nucleation in Some Normal Alkane Liquids. J. Chem. Phys. 1961, 34, 820–831. 10.1063/1.1731681.

[ref92] Günther E.; Schmid T.; Mehling H.; Hiebler S.; Huang L. Subcooling in hexadecane emulsions. Int. J. Refrigerat. 2010, 33, 1605–1611. 10.1016/j.ijrefrig.2010.07.022.

[ref93] You S.; Yu J.; Sundqvist B.; Belyaeva L. A.; Avramenko N. V.; Korobov M. V.; Talyzin A. V. Selective Intercalation of Graphite Oxide by Methanol in Water/Methanol Mixtures. J. Phys. Chem. C 2013, 117, 1963–1968. 10.1021/jp312756w.

[ref94] Huang L.; Günther E.; Doetsch C.; Mehling H. Subcooling in PCM emulsions—Part 1: Experimental. Thermochim. Acta 2010, 509, 93–99. 10.1016/j.tca.2010.06.006.

[ref95] Perepezko J. H.; Uttormark M. J. Undercooling and Nucleation during Solidification. ISIJ Int. 1995, 35, 580–588. 10.2355/isijinternational.35.580.

