# Supplementary material for: Nucleation and Supercooling Mitigation in Fatty Alcohol Phase Change Material Emulsions for Heat Transport and Storage
Source: ACS Omega. 2025 Apr 23;10(17):17705–16. doi: 10.1021/acsomega.5c00041 (PMC12059911; doi:10.1021/acsomega.5c00041)
Supplement: Supplementary file 1 — ao5c00041_si_001.pdf [file ao5c00041_si_001.pdf]

Supporting Information

Nucleation and Supercooling Mitigation in Fatty Alcohol Phase Change Material Emulsions for Heat Transport and Storage

Moritz Kick[1,2,]*,

Sebastian Gamisch[1],

Alexander Wittemann[2],

Monika Le[1],

Stefan Gschwander[1]

[1] Fraunhofer Institute for Solar Energy Systems ISE, Heidenhofstr. 2, 79110 Freiburg, Germany

[2] Colloid Chemistry, Department of Chemistry, University of Konstanz, 78457 Konstanz, Germany

*Corresponding Author

E-mail address: moritz.kick@ise.fraunhofer.de

Table S1: Selection of surfactants with HLB and purity reported by the manufacturer.

**Class**	**Surfactant**	**HLB**	**Purity**	**Supplier**
Linear	Laureth-2	7.3	>90 %	Sasol
Linear	Laureth-30	17.6	>70 % in water	Sasol
Linear	Ceteth-80	18.5	99 %	Sasol
Polymer	Poloxamer 407	18-23 (approx.)	n/a	BASF
Polymer	Inulin Lauryl Ester	n/a	25 % in glycerol	Ultra Chemicals

Table S2: Selection of nucleation additives with crystal data (SG=Space Group), particle size and purity reported by the manufacturer.

**Material**	**Solubility in oil**	**Crystal system**	**Lattice parameters**	**Particle size**	**Purity**	**Supplier**
C50-OH	soluble	Orthorhombic	SG: A2/a, Cell: a=9.27 Å, b=5.17 Å, c=266.45 Å, β=122.9 °[*]	Molecule	>80 %, PDI ~1.09	Sigma-Aldrich
C70	soluble	Orthorhombic	SG: A2/a, Cell: a=8.95 Å, b=4.97 Å, c=359.89 Å, β=122.8 °[*]	Molecule	n/a	Sigma-Aldrich
ZnO	insoluble	Hexagonal	SG: C_{6v}^4-$P6_3$, Cell: a=3.25 Å, c=5.21 Å[1]	20-30 nm	>95 %	Alfa Aesar
Carbon black	insoluble	Hexagonal	SG: P63/mmc, Cell: a=2,46 Å; c=6,71 Å[2]	460-500 nm	n/a	Scholz
CuO	insoluble	Monoclinic	SG: C2/c, Cell: a=4,68 Å, b=3,42 Å, c=5,13 Å, β=99,5 °[3]	30-50 nm	<=100 %	Alfa Aesar

[*]The lattice parameters of C50-OH and C70 were derived from an elongated C22-OH unit cell by Rietveld refinement of pure material XRD measurements.

Table S3: Emulsion formulations with 20 wt% C22-OH.

**Surfactant(s)**	**Surfactant concentration in wt%**	**DI-water conc. in wt%**	**Seed(s)**	**Seed conc. in wt%**
Laureth-2 + Laureth-30 (total HLB 16.6)	6	74	-<br>C50-OH<br>C70<br>ZnO<br>Carbon black<br>CuO	 0.04 – 0.84<br>0.04 – 0.84<br>0.04 – 0.84<br>0.84<br>0.84
Ceteth-80	2	78	-	-
Poloxamer 407	2	78	-	-
Inulin Lauryl Ester	2	78	-	-

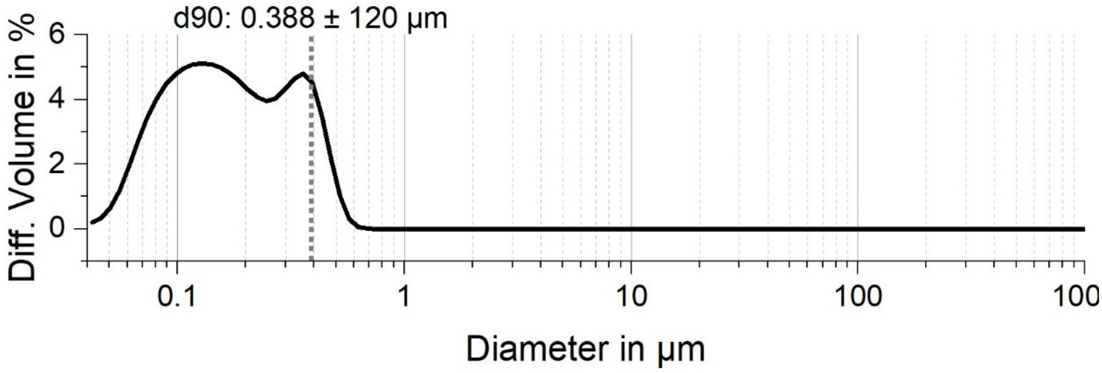

Figure S1: Particle size distribution of a stable 1-docosanol PCS, showing a d90 of 388 ± 120 nm.

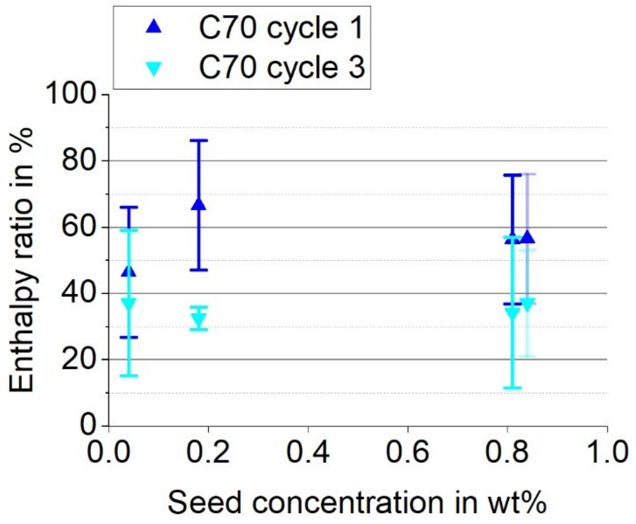

Figure S2: Decrease in enthalpy ratio over increasing number of thermal cycles, observed in PCS with C70.

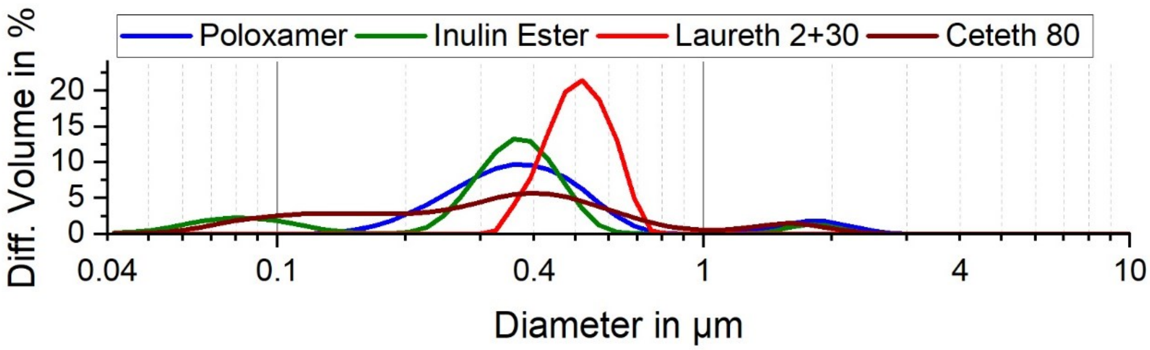

Figure S3: Particle size distributions of 1-docosanol dispersions with different surfactants, but without seed.
